# Dental Antiquities

**Published:** 1859-07

**Authors:** 


### DENTAL ANTIQUITIES.

### ARTICLE IX.

THE following curious article is extracted from an old book in the possession of the Editors of this Journal, entitled "ΜΙΚΡΟΚΟΣΜΟΓΡΑΦΙΑ. A Description of the Body of Man, Together with the Controversies and Figures thereto belonging. Collected and Translated out of all the Best Authors of Anatomy, Especially out of Gasper Bauhinus, and Andreas Laurentius. By HELKIAH CROOKE, Doctor in Physicke," etc. London, folio. 1618. [EDS.

### CHAP. XII. *Of the Sockets of the Teeth.*

IN the circumference or margent of both the Iawes, which place *Galen* called φάτνια, we meete with certaine cauities or hollow places which the Latines call *Alueolar* or *Locelli.* They are digged deepe that the Teeth like so many nayles might be firmlier fastned in them by *Gomphosis* or by way of Mortize; and therefore the rootes of the teeth are made iust fitte and answerable to the holes of the Iaw, for if they had bin broader the composition would haue beene too dissolute, and if they had beene too narrow the rootes of the teeth would not haue attayned to their bottomes, but being fitte as they are they are very firmely conteind. The number of these sockets can hardly be assigned, because they are sometimes single, sometimes double, sometimes treble, according to the variety of the roots of the Teeth.

Moreouer, although they be bony, yet they appeare to be almost like wax, and may well be compared to the holes of a hony combe which are often obliterated and often againe renued: for when a Tooth is drawne vnlesse a new one grow vp, the hole is so constringed that there remayneth no print thereof: yea if a Tooth fall out and a newe one doe rise vp in his roome, the former socket is obliterated and a newe one grauen for the new Tooth.

Hence *Falopius* concludeth, that the liuely and quickning Formatiue faculty remaineth in the Teeth vnto the period of a mans lifo, for by it they encrease and receiue their forme. So in the Cheekes and the Iaw when those Teeth which wee cal *Genuini dentes* or *dentes sapientiæ* doe shoote out, happily about seauenty yeares of age; yet euen then new sockets are made for them. Againe at fourescore when the teeth fall, the sockets grow vp and the iaw in that part is so compressed that the bone on euery side cleaueth together, and at length is vnited. For the sockets are not onely filled with a bony substance and rammed vp, but each partition growing to other do make a sharpe edge which in olde men serueth instead of teeth, if not to cut yet somewhat to breake and chew their meat. And so much of the Sockets, now we come to the Teeth themselues.

### CHAP. XIII. *Of the name, definition, figure, magnitude, number, site and articulation of the Teeth.*

The Teeth are called in Greeke ὀδοτες, as it were ἐδοτες of εδω, which signifieth to eate. In like manner the Latines call them *Dentes quasi edentes* of eating. They are the hardest of all the bones, hollow within, hauing small veynes, arteries, and nerues; articulated to the sockets or dens of their owne iaw *per Gomphosin*, or by way of mortize, fastened with membranes, and flesh, or ligaments, and primarily created for the comminution or mitigation or chewing of the meate.

That they are bones some men do deny, first because bones are insensible the teeth sensible. Secondly, because the bones haue certaine limits of auction or increase, neyther do they euer grow againe if they perish, but in teeth it is quite contrary. Thirdly, because they are harder then other bones. Fourthly, because bones exposed to the ayre do grow blacke, whereas the teeth keepe their whitenesse. Fifthly, they say, that *Hippocrates* in the 18 Aphorisme of the fift section distinguisheth them from bones where he saith, that cold things are enemies to the teeth, to the bones, and to the nerues. Finally, say they, there is a stone that will consume flesh called therefore *Sarcophagus,* which within forty dayes will deuour the whole body except the teeih. If therefore the teeth were of the nature of bones they also would be consumed.

We answere to the first, that sense is not of the nature of a bone. To the seconde, that they grow because iu attrition they are worne. To the third, that *more or lesse* do not change the species or kind, otherwise the spongy bones shold be no bones. To the fourth, that they are accustomed to the outward aer and haue no periostion on them, and the Philosopher saith, that that which is accustomable maketh no impression or alteration. To the fift, that is, to *Hippocrates* authority we say, that the bones and the teeth are affected by cold diuersly ; the bones onely by suffering, the teeth not only by suffering but also by sensation. To the sixt, first we may safely doubt of the experience; secondly, we may say that the teeth are not consumed because they are harder then other bones.

It remaineth therefore, according to *Hippocrates, Aristotle* and *Galen,* that the Teeth are bones, for saith *Galen* they cannot be referred to any other similar part, and therefore he placeth the teeth vnder the common *Genus* of the bones, and the rather because the qualities of their matter do agree, as hardnesse, solidity, smoothnes, whitenesse, &c. yet there are some differences betwixt them and other bones. For among all the bones none but these haue any exquisite sence because the teeth alone do admit nerues into their cauitie.

The teeth alone do increase as the life increaseth, that without any detriment they might performe their offices:

for being worne in the chewing of meates they are increased againe, but onely so much as they are worne away, otherwise they would soone fayle. The other bones haue no sence or but obscure, neither do they increase alwayes but when they come vnto their state or perfection they make a stay because they are not changed in the performance of their functions.

They differ also from bones because they are naked hauing no periostion without the, for then they would be payned in the wearing; hence it was that *Aristotle* doth oftentimes not number the teeth among the bones, but sometimes saith they are bony, sometimes that they resemble the nature of bones. And truly in their hardnesse, fastnesse, or solidity they doe exceed other bones, yea they are little softer than stones themselues, if they bee not allout so hard especially about their extremities. Some are stony like a milstone, others are sharpe like the steeld edge of a knife. They were made very hard that they might not weare so soone or be broken in the chawing or breaking of hard things, for they are not lined eyther with fatte or gristles as other ioynts are to hinder attrition. The teeth therefore do breake bones, resist the edge of steele, neyther can they easily as other parts of the body be burnt with fire. *Hippocrates* in his booke *de Carnibus* ascribeth the cause of their hardnesse to the quality of the matter out of which they are ingendered, for hee writeth that out of the bones of the head and the iaws there is an increase of a glutinous matter. In that glutinous matter the fatty part falleth downe into the sockers of the gums where it is dryed and burnt with the heate, and so the teeth are made harder then other bones because there is no cold remaining in them.

Their outward surface is by nature white, smooth and polished, but in age for want of care or by disease they become liuid or duskish. There groweth also vnto them a hard scaly matter by which as also by corruption they become rugged and vnequall, yet sayeth the Philosopher a horses teeth become whiter as he becomes older.

Their forme is before somewhat round, behind more plaine ; where they are ioyned one to another they are euen, and in their extremities sometimes thinne, sometimes sharpe, sometimes plaine, but alwayes vnequall.

They differ among themselues in figure, magnitude and number. Their figure in man differeth according to the difference of their vse in chewing. In fishes they are only acute or sharpe. In those creatures that chew the cudde they are of a double forme ; some *Grinders* and some *Shearers.* In men according to the three speciall diuisions of meates there are three kinds of teeth, *Shreaders* or *Shearers* called *Incisores, Dog* teeth called *Canini* and *Grinders* called *Molares.*

Againe mens teeth do not stand out of their formes as a Boares tuske, but are concluded or shut within the mouth ; neyther are they set like the teeth of a Saw as it is in dogs, for their teeth are giuen them instead of weapons.

The teeth a man are much lesse then the teeth of many other creatures lesse then he, for his mouth is much lesse : for according to the magnitude of the mouth is the strength of the teeth which consisteth in their figure, hardnesse, and quantity ; yea mens teeth compared among themselues are not equall but some greater some lesser, for the grinding teeth are greater than the rest.

The number of the teeth is not in all men one and the same, for some haue more, some have fewer, yet the more the better, for such, saith *Hippocrates* in the sixt section of the second *Epidemion*, are long liued, whereas those that haue few teeth are but short liued as *Aristotle* saith in the 3 chapter of his 2 booke *de historia Animalium.* The reason is, because the paucity and rarity of the teeth is an argument eyther of the want of spermatical matter or of the weaknesse of the formatiue faculty.

Againe, those that haue but few teeth do not chew their meate so thoroughly, nor prepare it so well for the stomacke. So that the Chylus is not so well concocted, and by consequence the bloud not so pure ; for the second concoction which is in the veynes of the Liuer doth not amend the error of the first concoction which is made in the stomack. Stories make mention of some men who haue had but one tooth in their vpper iaw, and therevpon haue some bin cald μονωδοντες as *Euripheus* the *Cyrenian*, *Euriptolimus* of *Cyprus* and *Pirhus* the King of the *Epirots*. Some in stead of teeth haue one continuall bone, such was the sonne of *Prusias* King of the *Bythinians*. Some haue had a double row of Teeth, as *Dripetinus* the sonne of King *Mithridates*, *Trimarchus* of *Cyprus*. Some haue had 3 rowes as *Hercules*, for so *Cœlius Rhodiginus* reporteth in the third chap. of his fourth booke. But for the most part there is but one row which is called φραγμὺς, *Septum* the hedge, because it hedgeth in the tongue. In both iaws there are in growne bodies 32 Teeth, 16 in each iaw, in some men 30, sixteene in the vpper iaw and 14 in the neather; in some 28, which is the least number, and then the foure last are wanting, for they doe not breake out in all men nor at the same time, sometime lying imperfect within their sockets, sometimes not formed at all by Nature.

And truly the grinding Teeth are the cause why Authors do not agree in the number of the teeth, for the nũber of these *Grinders* is often changed, the *Shearers* and the *Dog*-teeth in man very seldome. These Grinders are sometimes fiue in a side, sometimes foure, sometimes foure on the left hand and five on the right, or on the contrary; or else four below and fiue aboue, which variety is made by those Teeth which we call *Genuini*. The Teeth were made many not one, that they might do seuerall offices in cutting, breaking, and chewing the meate; againe, that when one is payned or perished eyther by force or by corruption the affection should not become common to them all.

A mans Teeth are set in both parts of his mouth in the vtter compasse of both the iaws; Fishes haue their Teeth in their Pallate and their tongue: Crabs in their stomacke.

*Alexander Benedcitus* witnesseth that in a mans pallat sometimes Teeth haue been ingendered, and *Eustachius* testifieth the same thing from his owne experience in a woman of *Rome.* Those creatures that chew the Cud haue their lower gang of Teeth whole, which reacheth even to their Lippe, so disposed and so equally ranked, that sayth *Galen, Nature is much to be commended for so ordering the dance,* so he calls their rank or course.

The Teeth therefore are streight, inclining to neither part, before are the *shearers;* called *Incisores,* next to them the Dog-teeth called *Canini,* and then follow the *Grinders* or the *Molares* which are the hindmost, yet so that they are all disioyned with a deeent and conuenable distance, which the Grecians call αρμιον, for they are set close one to another, and touch at the top, least that which is chewed should remaine in the spaces betwixt the and there putrisie.

The rootes of the Teeth doe enter into the holes or sockets of the iaws, to which they are so fitted, that a nayle sticketh not faster in a post then they do in their proper places, euery tooth being compassed with slender processes of the iaws which hould them fast that they fall not out or be shaken.

Euery Tooth also sayth *Galen* hath a nerue, and that nerue helps to establish it, for when the nerue is loosened with too much moysture, the Tooth becomes prominent and mouable: beside they are very strongly tyed by Ligaments which pass betwixt their rootes and the bottom of of their sockets. The Gummes also doe establish the teeth very much, fastning about them very exactly; and this *Commissure* of the Gums and the colligation of the iaws, *Rufus* cals *habena* or the bridle. In a word the Teeth are vnited by the interposition of nerues, membranes and flesh. The nerue is inserted into the cauity and so the Teeth may be said to be ioyned by *Synneurosis.* The fibres of the membranes do cleaue to the rootes of the Teeth: the flesh compasseth them about, which being eaten away, they eyther grow loose or fall out, and so they are ioyned by *Synsarcosis.*

Hence we may gather that the articulation of the teeth is by *Gomphosis* or by *mortize* which is a kind of coarticulation, and yet *Galen* doubteth hereof and saith, that their composition commeth neare to a Coalition: neare; it may be, but indeed they are coarticulated, which may be prooued, because when for want of nourishment they grow drye and begin to wither, then their coniunction is not so stedfast but loose and vncertaine, which hapneth also when the nerue under them is ouer moyst. Finally, their rootes are simple, double, treble and fourfould.

### CHAP. XIIII. *Of the Shearers and Dog-teeth.*

We sayd before that according to the three kinds of diuisions of Meats, there are also in men three kindes of Teeth called *Incisory*, *Canini* and *Molares*. *Incisory* we cal the *shearers* or the *shreaders;* they are seated in the forepart of the iaw, and because they are the first, they are called by *Hippocrates* and *Aristotle* *πρόσθεοι, primores.*

They are sharp edged like a knife and broad also, the better to divide that that comes betwixt them, and are therefore called *τομεῖς.* Sometimes they sheare a thing asunder as a paire of pinsons doth a wire, and that is when the lower teeth ar vrged, not against the ends of the vpper, but higher toward their bodies. And because these Teeth in laughter discouered, therefore the Grecians call them *γελάσιγοι.*

These *Shearers*, are for the most part foure, and those two that are next the *Dog-teeth* are lesse then the other, and shorter then the *Dog-teeth.* *Gorreus* sayth that some men in stead of these foure, either aboue or below haue onely two, but those so broade that they take vp the roome and performe the use of foure. Some againe, in stead of foure *shearers* haue sixe, eight, or more placed without any order, some of them being right, others crooking outward to the Lip, others inward into the mouth, which are so offensiue both in chewing and in pronunciation, that we are constrained sometimes to draw them, sometimes burne them, sometimes to file them. *Bauhine* maketh mention of a Doctor of Diuinity, whose two foreteeth in the lower iaw were double, for when the two vtter. were almost consumed about the fortieth yeare of his age two other grewe vp within them. The part of these Teeth which standeth without the Gummes is narrow betwixt the Basis and the sides, but before and behind thicker, growing thinne and broad by degrees vnto the extremity: especially it is so in the vpper two which are called *duales* and *Palæ*, because they are very like vnto a pale: the superfices is on the outside somewhat gibbous, and againe a little hollow on the inside. The other part of these teeth which is infixed in the iaw is toward the side flatted or compressed and endeth in a sharp point; for their roote is but single; yet greater for the most part, at least as great as any the roots of the great teeth: hence it is, that they easily fall out, especially those in the vpper iaw. In the extremities of these Teeth when men are growne to some age, there is either no perforation at all, or if it be it is wonderous obscure.

Next to these are the Dog-teeth called *Canini*, by *Aristotle & Galen* χυνοδοντες not so much for their figure, or because they stand out of the gummes as Dogs teeth do, as for their vse; for in dogs after the shreading teeth there are too kindes of sharpe teeth; some standing outward, others of a triangular forme. But a mans dog-teeth differeth from them both. They are onely two in each iaw, because a man is a mild and ciuil creature, whose strength consisteth more in wisedome then in fortitude of bodye, and therefore is not full of Dog-teeth as other furious and rauening creatures are, for to breake that which is hard two were sufficient. These go betweene the sharpe teeth and the plaine, the Shearers and the Grinders, and below at the gummes are broad and thicke, aboue sharpe and somewhat prominent beyond the course or order of the rest of the teeth. From the Shearers heerein they differ, because they are thicker and their head somewhat narrower but not so sharpe. They are also harder, that if any thing by chance do come betwixt the teeth which the shearers cannot diuide, these may breake it; for the shredders were made to cut meate short, and these to breake that which is hard.

Their roots as those of the shearers are but single yet infixed deeper, stronger also and lesse pressed or narrowe before than behinde. These rootes are longer then the single roots of the rest of the teeth, yet so that the vpper are sharper and longer then the lower. These they call the Eye-teeth, not that they are so produced that they touch almost the lower circumference of the eye, for they do not go aboue the lower end of the wings of the nose, but because a portion of the Nerue which mooueth the eye is derived vnto them; and hence it is that it is commonly and truly said, that to draw the eye-teeth is not without danger.

### CHAP. XV: *Of the Grinding Teeth.*

THE great Teeth the Grecians call, μύλαι OI μύλοι *Molares*, because by them the meate is broken as corne is broken vpon a Mill. They are seated in the inner part of the mouth and hidden by the cheekes: the reason of this position is, least the meat already shred or broken and by the tongue rowled vnto these Teeth to be further ground should fall out of the mouth. In their vse therefore and likewise in their forme they are like vnto a Corne-quern, vnequall, rough, hard, broad and great: Rough and vnequall, for so they are more fit for comminution, and that is the reason why Millers when their stones are growne smooth do pick them anew. But these teeth in olde men by long attrition do become equall and smooth and by reason of their perpetuall use, are more sooner broken and eaten out then the rest.

Wherefore that part of these grinders which standeth without the gum is foursquare, and that square more perfect in the 3 hinder teeth: and though their tops are very large insomnch as *Ruffus* in the eight chapter of his first Book calleth them τραπέζαι *Tables,* yet they are not smooth, bnt euery one, saith *Eustachius,* hath a pit one or two in the midst: whence it cometh to passe that their extreame or outward partes are somewhat high. Those two that are next the Dog-teeth are heightned on the inside and on the out-side, yet the first especially in the lower iaw doth sometime want his inwarde eminence: the third protuberateth or swelleth vp in foure corners; the fourth and the fift for the most part haue two eminences on the outside, on the inside but one. These cavities and protuberations meeting iust one with another do hold the meate faster that it fals not so easily from betwixt them.

They are the hardest, saith *Vesalius,* of all the bones, that they might not so easily weare away; broad also and plaine the better to leuigate the meate that is already shorne and broken by the other teeth; for it was not fit that a man should swallow his meat before it were perfectly ground: besides being so ground, the heat and moisture of the mouth assisting, there is made in the mouth a rude inchoation or beginning of concoction. They are called γομφίοι, not because their insertion is by way of mortize, for that is common vnto all the Teeth, but κ ϖὸ τῆε γομφίασεϖς, that is from the rough superfices of a Mill-stone, whose figure and vse the vpper partes of these grinding Teeth doe elegantly imitate. In Magnitude they differ, for the middlemost are the greatest, and those on each side lesser. The third is the greatest of all in Men, in Apes the fourth and the fift; for it was not fit that the internall capacity of the mouth which groweth narrow as the anterior part doth, should haue teeth so great as the middle where it was broadest of all. Sometimes to the fourth or the fift (but in the vpper iaw rather than the lower) there is adioyned as it were a halfe tooth, for Nature contending to make more grinders, is hindered by the narrownesse of the place, and therefore is constrained to ioyne them together.

Their Number is commonly in the vpper and lower Iaws on each side fiue ; that is in all twenty. The reason why there are more grinders than shearers is, because Nature doth vse according to the largenesse or narrownesse of the fissure of the mouth to allow creatures more or fewer of this kinde. Man whose mouth is verie narrow, and whose teeth were especially giuen him to chew his meate, hath therefore more grinders then shearers or dog-teeth : on the contrary in rauenous creatures there are more *shearers* then *grinders,* because they vse them as weapons. Hence it is, that in men there is little difference in the number of their Shearing or Dog-teeth, but of Grinders there is great variety : sometimes fiue, sometimes foure, sometimes six. *Eustachius* did neuer obserue so few as three, sauing once in a Cardinall. It may be his Iaws were short. And this variety *Gal.* makes mention of, although there be no such variety in Apes; whence saith *Eustachius* (and the obseruation is very good) it may well be concluded, that *Galen* did vse to cut vp not apes onely but men.

The two latter grinders both in site and generation, are called by *Hippocrates* in his Booke *de Carnibus,* τωφονεῆρες, *the Teeth of wisedome,* because when they growe vp a man beginneth to be wise : for they arise about the fourth Septenary, that is, at 28 yeare old, at what time men are or should be temperate and moderate. In the breeding of these teeth there is sometime great torment and paine, and therefore *Auicen* calleth them the teeth of Sense. They spring vppe sometime in olde age, and therefore *Aristotle* calleth them χρακτῆρες, because they perfect the age or growth : the Latins call them *Gemnuos,* to which Teeth this is peculiar, that they do not begin to breake out till all the rest haue beene a good while perfect. Sometime also they onely perforate the gummes but rise little aboue them ; sometimes they lurke altogether in the iaw, and are couered with the gums, sometimes they are not created at all.

The roots of these Teeth are perpetually of one forme in Apes, as *Eustachius* elegantly declareth, for in them the grinders of the vpper iaw haue three roots: of the lower iaw two, excepting sometimes the fift; the fourth as it is the greatest, so are the roots thereof greatest. In Men their roots are not alwayes of one forme, yet mostwhat they are many and alike: sometime in the fift of the lower iaw, (in the second also *Eustachius* observed the same) which because of his magnitude a man woulde thinke should haue many roots, there is but one, thicke and perforated with one broad hole. Other roots of the Teeth haue an equall or or vnequall superficies, and within two or three holes skilfullie thrilled and disseuered by thin scales, much like a Hony-combe. In the fift grinder there are three; in most of the rest two. Oftentimes the roots grow together, sometime they are separated, sometime a man cannot perceiue so much as the line that parteth them.

The Figure also of these Rootes is diuers, for some are round, others streight and acute, others obtuse, plaine and crooked. They differ also in magnitude, the roots of one tooth being thicke sometimes, sometimes narrow, of another short, of another long, of some broad and thin, yet for the most part the roots of the vpper teeth are longer and of the lower teeth shorter.

They varry also in situation, some touch one another, some growe neare, but touch not, some straddle one from another like the feete of a stoole. Againe, some stande off one from another, but yet are so incurued that their extremities doe touch, others running foure wayes are in their ends most of all disioyned, and these in drawing of Teeth are sometimes broken and put the man to cruell torment.

If there be three roots, especially in the vpper iaw, one runneth inward and two outward. To conclude, commonly the two vpper grinders which are next to the *Dog-*

*teeth* are fastened into their sockets with two roots, the other three with three. Sometimes the fift hath foure which is rare. Againe, the two lower Grinders which are next to the Dog teeth haue but one roote, the three others two. Those Teeth which we call *Genuini* or *Teeth of wisedome*, haue very short rootes, partly because that portion of the iaw wil not admit admit a deepe insertion, partly because in the leuigation or chewing of meate they are not in so much vse as the rest. Wherefore the rootes of the grinding Teeth of the lower iaw are fewer and shorter than those of the vpper, because the substance of the lower iaw is harder and more solid, and therefore better able strongly to containe the Teeth, and to bear their waight; but in the vpper iaw which is more rare and soft and wherein the Teeth hang, and therefore are more subiect to fall out with their owne weight, their roots are longer the better to fasten them, for they needed more ties as it were to binde them and to containe them within the iaw.

Hence it is that where the rootes are shorter the Teeth are drawne with lesse danger, but with more labor where they are many, especially if the rootes grow vnto the sockets.

The rootes of all the Teeth are perforated euen into their internal cauities, yet this perforation in perfect and growne Teeth is very small, and that in the sharpe pointe or top of the roote, through which a veyne, an artery and a nerue are admitted; of which Vessels we will speake in the next place but briefly because we have touched upon them before.

### CHAP. XVI. *Of the Vessels and Sense of the Teeth.*

Through the holes of the rootes of the Teeth al manner of Vessels do run and are diuersified in their internall cauities; veynes to bring them plentiful nourishment. Now it was necessary that the Teeth should alwayes grow, because in mutuall attrition they were one against another, and this we may certainly conclude by our owne experience. For if a tooth fall out or be drawne, that which groweth opposite against it will be longer than the rest of the Teeth of his own ranke, because it is no more worne by the Tooth that before was opposite against it, the rest of the Teeth by their mutual attrition in chewing of the meat are impaired and againe so much increased.

Arteries also do enter into the Teeth to preserue and keepe their naturall heat; hence it is that in inflamations there is a beating or pulsing payne felt, such as fleshy parts feel when they are inflamed. And this *Galen* first of all men obserued in the 8 chap. of his fift book *de Compositione medicamentorum, secundum loca*, for he found in himself not only paine but a pulsing paine in his Teeth, whence he concluded that there was one paine in the Gums, another in the very substance of the Tooth. Yea without any inflamation of the Gums sometimes there is paine in the proper body of the Teeth, sometimes in the nerue.

And againe vnlesse vnder the Tooth there were an artery, how could so much florid and pure bloud issue when the Tooth is perforated, which *Eustachius* observed in a man that fell into such a flux of blood that he had like to haue died vpon it.

They have also nerues soft and slender, which come from the third coniugation commonly so called, and runne through their rootes into their cauity, euen from their first conformation; where they are disseminated and their small surcles are mixed with the mucous matter of the Teeth; hence it is that when that mucous matter is become bony yet it easily consenteth with the nerues and the Teeth become sensible; whereas to other bones there are no nerues at al conuayed which may be dispersed into their cauities. And therefore it is truly saide that bones want nerues although the membrane which is called *Periostium* haue sense and nerues in it. Wherefore when this *Periostium* which is of exquisite sense is affected, the paine may be thought to be in the bones themselues when it is onely in the membrane; so in a Cicatrice which hath no sense wee imagine wee feele paine when indeed the paine is onely in the neighbor parts; and the membrane which inuesteth the Liuer being affected we thinke we feele paine in the Liuer whereas the *Parenchyma* or substance of the Liuer it selfe is insensible: vnlesse any man will say that the bones haue sense by the helpe of the Sensatiue soule by which they subsist: and yet that very obscure, because the hardnesse of the bones doth very much resist any such sensation; and thus we may say that bones do differ from plants as being particles of a sensatiue creature.

These nerues of the Teeth are very small, yet in our Table we haue made thẽ large immitating therein *Vesalius* and others. But by what order and manner of dissection, we vse to make demonstration of the vessels and nerues of the Teeth, saith *Bauhine*, wee will now relate as we learned it out of *Eustachius*.

These vessels are better demonstrated in the iaw of a great creature, as for example, in an Oxe, then they are in a Man: and againe the administration is easier in the lower iaw, then in the vpper. We take therefore the lower iaw of an Oxe and open it on the inside, & presently we meet a cauity full of marrow, together with the nerue inuolued in a membrane; when we haue remoued the marrow & slit the membrane throughout his length, then may wee perceiue the nerue made as it were of many strings, betwixt which do run propagations of veines and arteries. Moreouer, if you remooue this membrane with the surcles of the nerues and vessels a little vpward from the bone carefully that you break them not, you shall perceine some fibres distributed from the membrane not vnlike the Lawny threds of a cob-web. In like manner in the iaw of a Ramme certaine fibres do penetrate the bony partition which is betwixt the Nerue and the Teeth, but these fibres are most conspicuous at the roots of the grinders.

From these *Grinders* vnto the *Dog-teeth* and the *Shearers* there is a nerue conuayed accompanied with an arterie:

the nerue is deuided into two ; one part of it, through a hole in that place, breaketh vp at the lower Lip, and a branch of it runneth along vnto the *Shearers*, and affoordeth a small surcle to euery one of them ; another portion of it is ioyned with the vtter part of the rootes : the second part which is also the slenderest pearceth into the cauity of the teeth, and may euen without any greater difficulty be discerned euen in men.

And truely it is a strange thing that the *Shearers* and *Dog-teeth* which are the lesse and haue but one roote, haue notwithstanding allowed them great and conspicuous surcles of vesselles attayning to their insertion by a broade and open way ; whereas the Grinders which are the greater and haue three, sometimes foure rootes, are allowed but small and capillary surcles made of the former, doubly, trebly and foure-fold deuided, and creeping obscurely into their insertion.

Againe, if a *Grinder* or a *Shearer* bee gently and by degrees drawne out of his socket, you shall finde to arise with it out of the cauity of the Iaw very small fibres which are ioyned to the roots of the Teeth : you may also obserue that the parts of the bony partition are full of a mucous substance which is not vnlike to that whereof the teeth and their huls or huskes are generated.

Againe, when the Tooth is drawne, in the extremity of his roots you shall find a matter partly mucous partly fibrous, which carrieth a resemblance or shew of a nerue, a veine and an artery.

But if you deuide the Tooth in the middest you shall finde a mucous substance wouen with vesselles and some fibres : but these things may be better seen in the iaw of an Oxe or of a Ram then of a Man ; and yet euen in a man diligent search will find them out, though they be not so perspicuous.

Wherefore who can deny but that there is a Pulsatiue or beating payne euen in the inner part of the tooth, as *Galen* and *Eustachius* haue witnessed, when he shall perceiue an arterie and a nerue attaine thereinto? For veines, why should we not likewise beleeue that they enter into the Teeth, when wee see it sprinkled with bloud in men? and in Oxen may manifestly perceiue the vessell it selfe? for it is in the tooth as it is in that coate of the eye called *adnata*, as long as a mans Eie is well disposed the veines therein are not visible, but then onely become conspicuous when it is inflamed.

Concerning the Sense of the Teeth, the opinions of Anatomistes and Physitians are very diuers. Some thinke they haue no sense at all because they are bones and may be filed without paine; others thinke they haue Sense, but that of themselues without nerues, as other bones haue: and these men imagine that the paine of the tooth is without it, that is, in the membrane which compasseth the socket: others thinke they liue and haue Sense by their inbred heate. *Aristotle* determines that they feele cold sooner than heate, and are more affected by it: *Galen* saith they are pained, they beate and receiue soft nerues. But the question may be asked what part of the tooth hath this Sensation? *Varolius* answereth the body of the tooth, but on the inside onely. Others say that the whole Tooth indeede hath Sense, but the whole Tooth doeth not feele paine; it perceiueth the first and second qualities, but the first qualities onely doe paine it, because they exercise their power vpon the rootes of the teeth, into which certaine small nerues doe penetrate. This was *Archangelus* his conceit, and *Laurentius* hath almost the same. The whole Tooth sayeth hee doth *feele*, but more exquisitely on the inside more dully on the out.

Another question may be asked whence comes the sensation that payneth them? some answere, that it is by reason of the nerues and of their proper substance, so saith *Actuarius*. *Falopius* thinkes the paine comes by reason of a thin membrane compassing about the inner cauity. Others ascribe the cause to the nerue and the membrane ioyntly: others to the nerue which cleaueth to the neighbor partes and to the rootes of the Treth onely. Others to the nerue that entreth the Tooth and a membrane that cleaueth to the roote thereof as it were a *Periostium.* Others to a nerue which penetrateth tho substance.

These and such like are the diuers opinions of Anatomists concerning the sense of the Teeth. *Bauhine* interposeth his opinion on this manner.

The teeth, saith he, do feele, and the faculty of sensation is communicated to their substance by the mediation of a very thinne membrane which compasseth the inward cauity of the Tooth lightly hanging vnto it, and also of a soft nerue which attaineth into the same cavity: thus the Teeth have a proper kind of Touching which we cannot in words so well express as by instance. For vpon the eating of sowre or sharpe meates the Teeth are affected with a kinde of stupor, and then we commonly say our Teeth are set on edge, which kind of sensation is proper only to the Teeth and Gums, and is nothing else but a Symptome of the touching faculty.

But we must conceiue that each part of the Tooth is not equally sensible, but that the inside which is nearest to the nerue and the membrane is of quicker apprehension then the outside, for the outside partly because of the spisse and hard substance which like a shell couereth the inward part of the Tooth, and doth not admit the power of the nerue or the impression of the animall spirit: partly because it is continually accustomed to the mutations that come from the ambient ayer is not so sensible. Euen so we see that the callous skinne in the hands and feete of a laboring man is almost altogether without sense, thogh the skinne it selfe naturally disposed to be very sensible.

Wherefore that part which is without the Gummes or the shell of the Tooth is not ffended by hard and rough things, for though it be cutte or filed, or burnt with hot yron, yet it is not payned neyther is there any sense or signe of sense following therevpon, especially the height or top of the tooth which was made solid to breake the meate, & therefore the cauity not attaining so far the nerue also commeth short of it.

The reason why a part of the body of the tooth is not payned by fire or by hot iron, *Articœus* sayth is knowne onely to the Gods. As for vs we can say nothing but that it is a peculiar to the teeth that they are not equally affected eyther by all things that make alteration in them, or by those things which might offend them; but are more affected by heat and cold then by any other qualities, & by cold more then by heate, yet to giue some satisfaction, we say that the teeth are not offended by moist or dry by soft or hard thinges because their qualities are not so suddenly communicated to the membrane or to the nerue. But they are affected by hot and cold things because the animall spirits which are contained in the substance of the tooth and diffused through the same, are thereby altered; for those actiue qualities do pierce on euery side, affecting and changing together with their substance the animall spirite and by succession also the membrane and the nerues. And truly it is very reasonable to imagine that in the substance of the teeth there are great store of animal spirits more thē in other bones, first because they receiue soft nerues into their cauities, and againe because their internal substance is somewhat rare, made of a mucous matter condensated or thicknesse.

Obserue also that the paine which is felt in the substance of the tooth differeth much from that paine which is in the Gummes, eyther from their distemper or from any fiux of humor into them, and from the payne which is felt in the nerue that runneth vnder the roote of the Tooth.

The vse of their sensation is thought to be, first that being exposed to outward iniuries, and hauing no *Periostium* to compasse them about; it was fitte they shoulde haue an ingenite principle of Sense that they might bee able to discerne betwixt that which is profitable and hurtfull.

Againe, if we will beleeue *Galen* in the second Chapter of his 16 book *de vsupartium*, as all the others parts of the mouth, so likewise the teeth doe after a sort discerne of sapors or Tastes, and for that purpose they receiued soft nerues. For as the Skinne hath Sense, yet through the cuticle or scarfie-skinne which is not sensible; so the marrow of the tooth is apprehensiue of tactile qualities through the bony part, like as the neruous membrane which is vnder the nailes doth feele heat and cold through the nailes which haue no sense at all. And so much shall haue beene sufficient to haue spoken of the Sense of the Teeth. Now we come vnto their cauities.

### CHAP. XVII. *Of the inward cauity and Membrane of the Teeth.*

Wee saide before that the Vessels and the Nerues did enter into the cauities of the Teeth, and therefore it would be very fitte we should acquaint you what this cauity is.

All the Teeth are hollow on the inside, but this cauity is greater or lesser according to the magnitude or figure of the Teeth, which thing also *Aristotle* acknowledged in the 7. chapter of his 3. booke *de historia animalium*, where hee sayeth that the teeth are bones partly concauous and partly solid. Notwithstanding some new Anatomists haue been so impudent as to challenge to themselues the inuention or finding out this cauity. *Galen* indeede in those bookes of his which are extant, doth not write how great or what a kind of cauity this is, nor yet what is contayned therein; yet because ofttimes he makes mention of the *Dennes* of the Teeth, it is not likely that he was ignorant of them; for if hee had beleeued that the teeth in their first originall had had no cauities, it had bin an idle thing for him in vehement payne of the Teeth to commaund that their solid substance should be perforated with a small wimble or piercer, in the ninth chapter of his 5. booke *de compositione medicamentorum secundum loca.* Againe, *Hippocrates* in the 4. *Epidemium* reporteth that a Childes Teeth were eaten with a corroding vlcer, those especially that had cauities or were hollow, whereby *Hippocrates* doth insinuate, first that the substance of the Teeth may bee corroded, and secondly that they haue a natural cauity in them.

This cauity is in children of seauen years old and somewhat vpwards very large, and circumscribed with a thinne scale very like vnto a hony-combe. It is also full of a white humor, not of marrow, such as we may see in the sweet-tooth of a Calfe when it is broght sodden vnto the Table, but this humor in processe of time is dried, becomes harder, and for the most part turnes bony, and so the cauity is euery day diminished; yet so that in the middest at the roote there remayneth alwaise a *Sinus* which scarcely reacheth aboue the height of the Gummes: for it was necessary there should be an empty space left, because of the insertion of the vessels and the dilatation of the artery: yet some men affirme that the pulsation wee feele in our teeth doth not proceede from the beating of the artery, but from a spirit or ayre moued, as sometimes we finde it to be in our eares.

These things sayth *Eustachius* are best perceiued in the *Grinding* teeth of an Oxe broken; and in a Calfe and a Lambe each *grinder* hath three cauities, one *anterior* another *posterior* and the third in the middest; and the middlemost cauity is perforated with a crooked hole like the letter C which reacheth as farre as to the top of the Gummes: in the two other cauities there is a mucous matter contayned, which for the most part as the Tooth growes perfect, turneth bony. Beside, because in these creatures we find oftentimes two, sometimes three, sometimes foure grinding Teeth ioyned together, Nature to make a mutual consent between them, hath perforated the bony partition with a transverse hole, through which a matter like a small membrane passeth out of one cauity into another, as surcles of vessels do passe through their perforations.

This cauity is compassed with a membranous substance, which *Falopius* and *Laurentius* call a thinne membrane. *Goreus* saith it is a production of the *Piamater*. *Colvmbus*

esteemeth it to bee made of a complication of veines, arteries and nerues, which imbibeth or sucketh vp the matter that falleth vpon it, from whence come the greatest and most intolerable paynes of the tooth-ach.

And indeed this membrane is of most exact Sense, and by it the Teeth are so apprehensiue of heate and cold: yea sayeth *Bauhine* wee consent with *Columbus* herein that the flux of humor out of the Brain vnto this membrane is the true cause of the greatest paines in the teeth which do so long endure as the humor is detained in the Membrane, or till the braine be purged, and so the cause of the flux be taken away.

By reason of this cauity if the teeth be perforated by an affluence of sharpe humors, which perforation doth reach to the cauity, then are the teeth quickly rotted euen vnto the roots, againe in this cauity, especially the erotion and putrifaction of the teeth doth begin. In it groweth that dolorous rottennesse, and in it sometimes are wormes gathered, which do miserably excruciate and punish the patient. The vse of the cauity is, that by it the teeth may be better nourished, and receiue the faculty of sensation.

### CHAP. XVIII. *Of the generation and use of the Teeth.*

CONCERNING the generation of the Teeth, there are diuers opinions; some thinke they are generated within the Wombe, as *Columbus* and *Eustachius*; some without the wombe, as *Aristotle;* some partly within and partly without, as *Hippocrates* who maketh a threefolde time of their generation in his Booke *de Carnibus*, of a threefolde Aliment, which ministreth matter unto them. The first is from the sustenance they receiue in the wombe; the second is after birth by the Milke which the child sucketh; the third is after he hath cast his teeth by the meat and drinke that he eateth whereby new teeth are engendered: for, saith he, whatsoeuer is glutinous in the Aliment that maketh Teeth, but the fatty part, which heere is more plentiful then in the matter of the rest of the bones, is ex-iccated by the power of the heate. So also, saith *Laurentius,* as this threefold kinde of Aliment differs in thicknesse, so doth the solidity, hardnesse and thicknesse of the teeth varye, for those teeth that are engendered of the Aliment which the infant vseth in the wombe, or when hee suckes his mothers brest are but soft and do easily fall away, but those that are made of more solide meats are also firmer.

The truth is that they are generated in the womb together with the rest of the bones, with which they are not delineated but formed and absolued by degrees: wherefore they lye for some time imperfect in the Iawes, neyther do they all breake their prisons at the same time, but some sooner, some later, according as the necessity of Nature dooth require. And this is the cause why some made a double time of their generation, one in the wombe, another out of the wombe.

In the wombe after the generation of the Iaws there are twelue Teeth formed, foure of which are *Shearers*, two *dog-teeth* and six *Grinders*, all which do want roots and lie hid in their sockets, on euery side compassed therewith, and the gummes whole aboue them. And this may be seene in the iaw of an abortive infant or other creature, yea if it dye presently after the birth; for if you cut vp the Iaw you shall finde Teeth therein. Some have bene borne with their Teeth out of their gummes, as of old time *M. Curtius Dentatus,* and *Cn. Papyrius Carbo.* Of later times, the same is reported of *Richard* Crooke-back the Vsurper.

The substance of the Teeth being yet imperfect is partly mucous and partly bony, for if you take away the husk of the Tooth, (for there is about every Tooth such a white, mucous and slimy substance somewhat membranous wherewith the tooth is couered, which also is so much the more mucous by how much the tooth is the softer and the younger) perforating it in the vpper part, that the end of the Tooth may peepe out, then shall you perceiue that the tooth is partly bony, partly mucous; for that part which was to rise aboue the gummes is fashioned into a white scale, thin and excauated or hollowed like a Hony-combe, and so the vpper part of the Tooth is bony, hard and hollow. The other part which should haue remained infixed in the Iaw, is soft, moyst and mucous, like the substance that is in a young quill.

This substanee seemeth to haue fibres and threds and to be couered as it were with a thin coate, for the superficies thereof is like to a smooth tunicle fastned and conioyned to the substance that it containeth: wherefore a resemblance of the generation of the teeth wee haue somewhat expressly in the generation of a quill: for the part which is without the skin is horny and hard, but that which is within the wing is softer and moyster, yea sometimes like blood or congealed Phlegme. This soft part of the teeth as they breake the flesh hardneth by degrees, and by degrees becometh bony. Sometime also it is hollowed within and formed into roots.

The hull or huske of the Tooth whereof we spake euen now serueth instead of a Ligament, for by it the tooth is fastned as with glew to the socket and to the gums.

In Infancy the Teeth be within the Gummes that they might not byte the Nurses nipple: the seauenth moneth they beginne to breake out, or later sayth *Hippocrates*, if the Infant do toothe with a Cough, and then they are troubled with Agues, Convulsions, Scowrings and such like, especially when they breede their *Dog-teeth*. *Galen* rendereth a reason, because when the Gummes are perforated by the Teeth the paine is as violent as if a goade were thrust into the flesh, but indeed the teeth are more paynfull then goades, for if a goade be once fastned it resteth, but the tooth issueth still to the extent of his augmentation.

The Teeth breake out not altoghter, but the vpper sooner then the lower, and the Shearers sooner then the Grinders: that they issue later in men then in bruite beastes (for the Elephant, saith *Aristotle* in the 5. chap. of his 2. book *de hist. animal.* breedeth his teeth as soon as hee himselfe is borne) the reason is saith *Aristotle* because a man aboue all creatures hath lesse of that earthy excrement whereof they are engendered· But because the first Teeth and those that follow them which are thought to bee regenerated, do lie hidden in the iaw bone, therefore wee rather say that the cause of their late yssuing in men is to bee attributed to the good will and pleasure of him that made them, who moderateth all things according to his owne wisedome; yet when the childe comes to chewing, that he might cease to be troublesome to his mother, and not lie alwaise lugging at her brests but fall to stronger kinds of meate, therefore at length Nature put them foorth; for euery particle is then accomplished when Nature standeth in neede thereof, and this is the reason why the Teeth are not formed till after the birth. For this cause also sayeth *Aristotle* the Shearers doe yssue before the Grinders, because the meate is first shred before it bee ground.

Dentition or the breeding of the Teeth begins about the seauenth yeare, sometimes sooner, but then saith *Hippocrates* in his booke *de carnibus,* they are ingendered of an ill humour. The first Teeth that arise are the fore-teeth; *Democritus* saith because their sharp ends make way before their due time. *Aristotle* reprehends him and giueth another reason, because that which is sharpe doth soonest grow blunt: and therefore Nature sendeth a supply of others; the broade teeth are not blunted at all but onely leuigated by attrition, and this is *Aristotle* his conceite in the 8. chapter of his fift booke *de generatione animalium.*

When the first Teeth about the seauenth yeare are either drawne or thrust out by those that come vnder them, then doe those first Teeth appeare soft and as it were hollowed: and therefore some have thought them onely Appendances of certaine rootes left in the iaw, of which roots, as it were of seede, a new hope or succession of teeth is broght forth. *Vesalius* therefore counsels vs, to take heed that when a childes tooth is broken by accident we doe not draw the roote, for then haply the tooth will not grow againe. But Anatomy teaches vs the contrary, that is to say, that there is no coniunction betwixt the imperfect teeth that fall at seauen yeares of age and the perfect that arise after. Nay they do not so much as touch one another, for there is a partition in the midst betwixt them before the new tooth can breake forth. And thus much of the first time of the generation of the teeth within the wombe.

The second time of their generation is without the womb, about the seauenth yeare, and these teeth are commonly thought to be Regenerated ; but to say trueth and to speak properl, they are then neither generated nor regenerated. For together with the first Teeth in the beginning of generation they doe receiue with the rest a rude kinde of forme, and are made of the same matter ; otherwise we must be constrayned to confesse, (and that were very absurd) that Nerues, Vessels, Ligaments and Membranes which are spermaticall partes and doe consummate the frame of the Teeth, doe beginne their generation after the Infant is borne seuen yeares, more or lesse. It is true indeed that these latter Teeth are not sooner absolued or perfected then in the seuenth yeare or there abouts, and the proportion seemeth not to bee much amisse, for they first breake out of the Gums about the seauenth month, and the second about the seauenth yeare. And this proportion *Hippocrates* in his booke *de septimestri partu* standeth much vpon, not only in the production of the Teeth, but also in other mutations in the body of man.

*Falopius* conceiueth that the latter Teeth are made of the same matter with the former by the seminary faculty which remaineth in the iaws, and *Eustachius* confesseth that if you remoue the bony partition that is betwixt the first and the later teeth you shall finde the seedes of the Teeth one vnder another, I meane of the Shearers and the Dog-teeth, but of the Grinders he neuer found any seedes, and yet he thinketh it reasonable that they should haue a rude originall in the wombe which is accomplished afterward at leisure.

The teeth which about the seauenth yeare break out, or as some say are renewed, are in eyther iaw ten, foure Shearers, two Dog-teeth, and foure Grinders or Maxillaries, that is two next vnto the Dog-teeth, and two that are called *Genuini* or *Teeth of wisedome.* The shearing teeth when they breake forth do thrust the first shearers out before them and issue betwixt the two first, the second, and the Dog-tooth that is next vnto them. But if the former teeth will not fall or be not pulled out, or if the latter issue before the first fal, then the latter worke their way through new sockets and turne in the vpper iaw outward, in the lower iaw inward, so that there seemeth to arise a new row of teeth, and this indeed hath deceiued many Hystorians and some Anatomists also.

The Dog-teeth also do fall out and the place of the succeeder is a little of the one side the roote of the former.

The reason why the teeth about the fourth fift sixt or seauenth yeares do grow loose, is because the sockets do continually increase and the teeth are but soft and therefore doe soone perish, because the harder Aliment which from thenceforth accreweth vnto them is nothing conuenable to their substance and then they putrifie and fall away: but those teeth that brake out at the seauenth yeare receiue nourishment agreeable to their substance, and therefore do continue as long as their nourishment is supplyed. Among the grinders the two first do sometimes thrust out their predecessors, but for the most part they arise at their sides and increase the number; those two that are called *Genuini* doe neuer thrust out them that were before them, but yssue sometimes in extreame old age, in the very ends of the iaws; yea *Aristotle* reporteth that these teeth haue arisen not without great paine after fourscore yeares. Notwithstanding this is more rare in men then in women. *Hippocrates* in his booke *de Carnibus* witnesseth that these teeth which grow vp so late doe wax old together vnlesse by mischance they fall out or perish.

The manner of the generation of the teeth *Fallopius* thus expresseth. The quickening faculty by an actiue spirit makes the bone hollowe ; at the same time is ingendered a membranous huske which hath two ends, one posterior whereat a small nerue, a veyne and an artery do meete. The other anterior whereat hangeth a neruous tayle like growne Malt : and this taile creepeth through a narrow perforation of the bone to the side of that tooth which hath a successor and so passeth vnto the Gums.

In the foresaide husk there gathereth together a white and slimy matter, and the first part of the tooth becomes bony when as yet the latter part is soft, euen so as we said it was in the teeth formed in the mothers wombe.

Euery tooth yssueth through that hole dilated, through which the tayle or beard of the huske was transmitted. Instantly the huske is broken and becometh as we said before a ligament to the tooth, and the tooth it selfe issueth naked and hard, notwithstanding the hardest part of it receiues a further perfection by induration of his matter without the Gums.

The primary and first vse of the teeth was to diuide to breake, to chew or mitigate the meate and so to prepare it for the stomacke. Againe, another vse of the teeth is for the forming of the voyce, for Shearing teeth are of great consequence to true pronunciation of letters or words ; and hence it is that those that want their teeth cannot so well pronounce R, S, X, Z, yea it is thought that the shearing teeth in men haue no other vse but only for elocution although the Infant doth not lightly speake before he haue all his teeth, or at least some of all kindes. VVhen he hath a few teeth he will mumble and as it were record, but cannot articulate plainely till he haue all, yet these shearers do not equally assist vs in the pronunciation of all words, only they make the prolation of some more cleare and facile. Those words that are formed of T and

R cannot bee pronounced without the shearing Teeth articulately, for they require that the Tongue should rest vpon the fore Teeth. *Laurentius* saith the Teeth were made for ornament, but *Homer* more wisely, that they were giuen to men to keepe their tongues within compasse. *Bauhine* thinkes that the Teeth of themselues are not onely no ornament but a fearfull sight, and therefore Nature compassed and distinguished them with Gums, couered them with the lip as it were with a buckler the better to breake and change the ingresse of the outward aer. Those creatures who are able eyther with their hornes to defend themselues as a Bull and Cow, or with their hoofes to offend their aduersaries, those creatures I say haue teeth only to eate withall. They that haue saw-teeth do both mitigate their meat by them and stand vpon their guard with them, for they are giuen to such creatures both to offend and to defend, whereas a man hath his hands to defend himselfe.

And thus much shall be sufficient to haue saide of the Teeth, their Sockets, their Nature, Figure, Magnitude, Number, Site, Articulation, Kindes, Vessels, Sense, Cauitie ; and finally of their Generation and Vse.

And thus we haue brought to an end the history of the bones of the Scull and of the Face. Another bone there is which belongeth not indeed to the head, but but much lesse vnto the Trunk or the ioynts, whose history we will therefore insert in this place because it is neare vnto the Head though it be not of it, and that is the bone *Hyois*.

